# Spatiotemporal changes of pelagic food webs investigated by environmental DNA metabarcoding and connectivity analysis

**DOI:** 10.1098/rstb.2023.0178

**Published:** 2024-07-22

**Authors:** Daniele Bellardini, Luca Russo, Viviana Di Tuccio, Daniele De Luca, Gabriele Del Gaizo, Gianpaolo Zampicinini, Florian Kokoszka, Vincenzo Botte, Francesco Colloca, Fabio Conversano, Pasquale De Luca, Daniele Iudicone, Francesca Margiotta, Simona Saviano, Paolo Vassallo, Daniela Cianelli, Domenico D’Alelio

**Affiliations:** ^1^ Department of Integrative Marine Ecology, Stazione Zoologica Anton Dohrn, Villa Comunale, Naples 80121, Italy; ^2^ DiSTAV, Department of Earth, Environment and Life Sciences, University of Genoa, Corso Europa 26, Genoa 16132, Italy; ^3^ PhD Programme in Evolutionary Biology and Ecology, Department of Biology, University of Rome “Tor Vergata”, Rome 00133, Italy; ^4^ Department of Research Infrastructures for Marine Biological Resources, Stazione Zoologica Anton Dohrn, Villa Comunale, Naples 80121, Italy; ^5^ Information and Environmental Reporting Area, Agenzia Regionale per la Protezione Ambientale del Lazio, Via Boncompagni 101, Rome 00187, Italy; ^6^ Sezione di Oceanografia, Istituto Nazionale di Oceanografia e di Geofisica Sperimentale—OGS, via Auguste Piccard n. 54, Trieste 34151, Italy; ^7^ Consiglio Nazionale delle Ricerche, Istituto di Scienze Marine (CNR-ISMAR), Naples 80133, Italy; ^8^ NBFC, National Biodiversity Future Center, Piazza Marina 61, Palermo 90133, Italy

**Keywords:** coastal system, fish, marine ecology, Mediterranean Sea, plankton, trophic interactions

## Abstract

Environmental DNA metabarcoding (eDNA metaB) is fundamental for monitoring marine biodiversity and its spread in coastal ecosystems. We applied eDNA metaB to seawater samples to investigate the spatiotemporal variability of plankton and small pelagic fish, comparing sites with different environmental conditions across a coast-to-offshore gradient at river mouths along the Campania coast (Italy) over 2 years (2020–2021). We found a marked seasonality in the planktonic community at the regional scale, likely owing to the hydrodynamic connection among sampling sites, which was derived from numerical simulations. Nonetheless, spatial variability among plankton communities was detected during summer. Overall, slight changes in plankton and fish composition resulted in the potential reorganization of the pelagic food web at the local scale. This work supports the utility of eDNA metaB in combination with hydrodynamic modelling to study marine biodiversity in the water column of coastal systems.

This article is part of the theme issue ‘Connected interactions: enriching food web research by spatial and social interactions’.

## 1. Introduction

Unlike on land, most primary and secondary producers are organisms smaller than a few millimetres in the ocean water column [1,2]. These floating communities belong to the plankton and tightly drive aquatic food webs [1]. Plankton have huge functional diversity, with autotrophs, heterotrophs, mixotrophs, herbivores, carnivores and detritivores coexisting at the microscale (up to 100 m) [3,4]. Such diversity displays unexpectedly long trophic pathways in a few cubic metres of seawater and feeds important categories of fishes [5], like those playing as ‘keystones’ in marine food webs [6].

By definition, plankton drift with currents [7], and their communities show high spatiotemporal variability owing to the tight interplay between environmental factors and water transport [8,9]. On the one side, local physicochemical conditions select for plankton organisms with different physiology, biological cycles and feeding behaviours [9,10]; on the other side, ocean currents can reciprocally segregate or mix different water masses with distinct physicochemical properties which plankton can benefit from, or not [8,9,11]. This variability quickly scales up to fish communities [10,12], whose distribution in space and time is shaped by both evolutionary and ecological factors, from spawning timing and migratory abilities to salinity tolerance and food preferences [13–15].

Pelagic food webs, spanning from plankton to fish, can be, therefore, highly dynamic in space and time, especially at the regional scale (1–100 km) and close to the coast [16], where riverine inputs and intensified water flows can profoundly modify environmental conditions and the ecological state of the water column [17,18], with cascading effects on fish populations and pelagic food webs [12,19,20]. In this context, an important question is whether the variability of plankton–fish consortia, whose predator–prey relationships are a fundamental factor for fish recruitment, is higher in space or time in coastal systems, posing fundamental implications in the marine ecological study and management of ecosystem goods and services, and economic activities like fisheries and fish aquaculture [5,21].

This article is a proof-of-concept for the study of spatiotemporal changes in pelagic food webs in a coastal system at the regional scale (Campania region, Mediterranean Sea) and across different seasons (2020–2021). The study integrated the plankton–fish biodiversity inventory with the environmental DNA technique [22] and coastal connectivity (CC) analyses carried out with Lagrangian modelling to assess the probability that ocean currents transport plankton from one site to another over a given time interval [23]. Such an integrated approach allowed us to evaluate the degree of ecological and physical connectivity among communities and geographical sites and to assess the relative contribution of space and time in pelagic food web variability.

## 2. Material and methods

### (a) Physical connectivity among coastal sites

To track estimated connectivity among nearshore sites we first performed numerical simulations using a regional ocean modelling system (ROMS) developed for the Tyrrhenian Sea (2 km resolution); then, the results of this first simulation were used as initial and boundary conditions for a finer grid model called Gulf of Naples Advanced Model (GNAM), covering the Campania coast with a 500 m resolution to obtain high-resolution output. GNAM is a free-surface, terrain-following, primitive equations ocean model widely used for a broad range of applications [24] and recently validated for the Gulf of Naples (GoN) area using a multiannual comparison with coastal high-frequency radar data and hydrological measurements [25]. We then used the ROMS velocity fields to run a Lagrangian transport package of virtual passive particles released along coastal areas, following velocity fields and constrained to fixed release depths (1 m).

**Figure 1.**
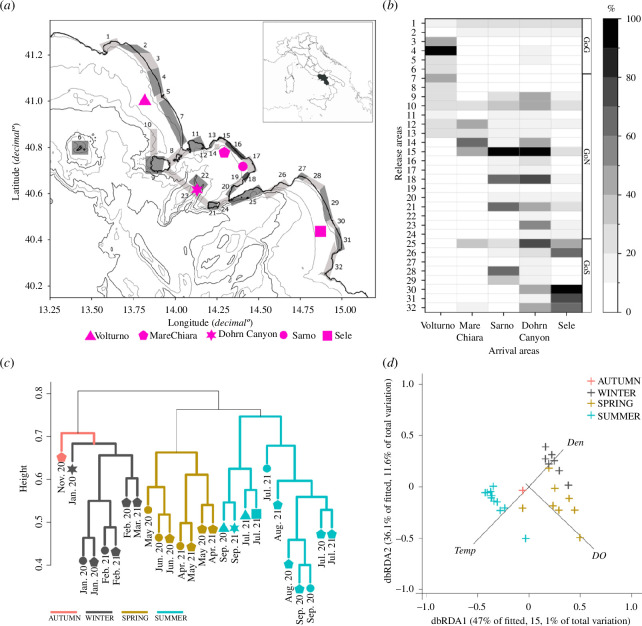
(*a*) Sampling map where numbers correspond to particle release areas (defined by the contiguous rectangles) along the Campanian coast; geometric shapes represent sampling stations. (*b*) Annual physical connectivity matrix showing particle migration rates, from release to arrival areas, as percentages (%). Cluster dendrogram (*c*) and dbRDA plot (*d*) based on Hellinger transformation of the reads count for V9-18S eDNA. (*d*) dbRDA plot fitted to significant predictor variables determined by BEST selection distLM (Temp, Temperature; DO, Dissolved Oxygen; Den, Density).

CC is defined as the percentage of numerical particles, representing small water volumes and the plankton therein, leaving a source site (*i*) and arriving at a destination site (*j*) over a time interval *t*. Given *n* different coastal areas, an *n × n* connectivity matrix was evaluated for each given time scale, where the (*i*,*j*) element was the fraction of the particles from source area (*i*) to destination area (*j*), in the released time (*t*). In this study, we released particles along the Campania region coast (figure 1*a*) every 5 days for 5 years (2013–2017, around 250,000 particles per year), and tracked them for 96 days (for IDs, the number of release areas and the seasonal connectivity, see electronic supplementary material, table S1 and figure S1). Finally, a connectivity network was produced by summing the particle fraction of the areas of the sampling sites. Connectivity networks were visualized with Gephi v. 0.10 [26].

### (b) Seawater sampling

We sampled environmental DNA (eDNA) onboard the *R/V Vettoria* between January 2020 and September 2021 in different sites along the coast of the Campania region (Southern Tyrrhenian Sea, Italy; figure 1*a*). Sampling in the GoN occurred at an approximately monthly scale; sampling at the plumes of three rivers in the GoN, Gulf of Gaeta (GoG) and Gulf of Salerno (GoS) occurred during summer (see electronic supplementary material, table S2). In the GoN, we sampled the long-term ecological research site MareChiara (DEIMS iD: https://deims.org/0b87459a-da3c-45af-a3e1-cb1508519411) (40°48′ N, 14°15′ E) [27], the Sarno River mouth (40°43′ N, 14°27′ E), and an offshore site localized above a canyon, i.e. the Dohrn Canyon (40°36′ N, 14°08′ E). Other stations were at the Volturno River (40°58′ N, 13°50′ E) and Sele River (40°28′ N, 14°55′ E) mouths, in the GoG and GoS, respectively.

At each sampling site, we collected surface seawater using Niskin bottles, we filtered 0.5–2 L of seawater on nitrocellulose filters (porosity 0.45 µm, diameter 47 mm, GVS North America, two replicates per each sample) that were flash-frozen in liquid nitrogen and then preserved at −80°C until further analyses. A SeaBird 911 Plus multi-parametric probe provided temperature, salinity, density, conductivity, dissolved oxygen, fluorescence and turbidity data.

### (c) Metabarcoding analyses

We extracted DNA using the E.Z.N.A. Mollusc DNA kit (Omega Bio-Tech) following the manufacturer’s instructions. Metabarcoding libraries were prepared using the primers Euk1391F and EukBr [28] for the V9-18S region and 12S MiFish_U forward (fw) and reverse (rv) [29], respectively, and sequenced by GenomiX4Life (Illumina MiSeq; https://www.genomix4life.com/it/) following published protocols [30,31]. The V9-18S region was chosen for its capability to detect most of the planktonic taxa, as already done in the GoN [32] and in other marine systems [33,34]. On the other hand, the 12S region was used only to selectively detect fish presence, since the number of reads referring to each amplicon sequence variant (ASV) did not allow for an estimate of the relative abundance of fish taxa.

Illumina paired-end V9-18S raw reads (FASTQ format) were pre-processed to generate ASVs in RStudio [35] using the dada2 pipeline [36]. Primer sequences were removed, and fw and rv reads were trimmed based on the quality score (the first 150 bases of each fw and rv reads were kept; the maximum number of ‘expected errors’ allowed in a read = 2; max number of ambiguities = 0). Filtered reads were used to train the error model from the data using a machine-learning approach. Fw and rv reads were then denoised to generate ASVs by applying the trained error model and using the option ‘trimOverhang = TRUE’ to account for the fact that the sequenced amplicon was smaller than the read size. Finally, fw and rv reads were merged and checked for chimeras. 12S ASVs were also generated with the dada2 R library; adapter trimming and preliminary filtering were instead performed using cutadapt [37] with the ‘linked adapter’ option in paired-end mode, allowing 20% mismatch, truncating 3′ bases when quality was <15 and discarding untrimmed reads. All reads with ambiguities were then removed in dada2 before read error estimation and denoising; denoised reads were then merged into contigs, allowing a maximum of nine mismatches, and finally checked for chimeras.

To account for differences in the number of V9-18S region ASVs across samples, data were normalized at the median value of reads across samples (*n* = 91,446) using the function ‘rrarefy’ of the vegan R package [38]. Taxonomy was assigned to ASVs using a consensus taxonomy approach through the Python script ‘taxonomy_assignment_BLAST.py’ (https://github.com/Joseph7e/Assign-Taxonomy-with-BLAST/blob/master/taxonomy_assignment_BLAST.py) from five BLAST hits, using a minimum coverage of 70% and assigning taxa to species if the percentage of identity was ≥99%, to genus if <99% and ≥95%, or to any other taxonomic categories if higher than such thresholds The script was run twice, the first time against the SSU eukaryotic rRNA database of NCBI (https://ftp.ncbi.nlm.nih.gov/blast/db/SSU_eukaryote_rRNA.tar.gz, last modified 7 December 2022) and the second time against the PR2 database v4.14.0 (https://github.com/pr2database/pr2database/releases).

### (d) Ecological data analysis

We performed statistical analyses using data without replicates and, where present, relative abundance was averaged, as done in similar studies (e.g. [33]), to a total of 26 eDNA samples. Environmental and biological (V9-18S eDNA) data were analysed separately (see electronic supplementary material, tables S2 and S3); the similarity matrices for environmental data were based on Euclidean distances of normalized data, while for biological data, we employed Hellinger transformation to reduce the impact of highly abundant taxa [39]. As the first exploratory analysis of beta-diversity, we conducted cluster analysis on V9-18S Hellinger-transformer data in the *vegan* package (‘*decostand*’, ‘*vegdist*’ and ‘*hclust*’ functions) [40]. To test for the presence of seasonal differences between samples, we performed a two-way permutational multivariate analysis of variance (PERMANOVA, *p* < 0.05) with the fixed factor ‘season’ (three levels: winter, spring, summer), followed by a PAIRWISE test for significant terms.

We examined relationships between biological and environmental variables using the distance-based linear models (DistLM) routine that models linear relationships between dissimilarity matrices of biological data and predictor environmental variable(s) [41]. This routine allows fitting one or more environmental predictors to one or more biological variables. Among the model-building options, we selected the ‘Best’ procedure for the variables selection and ‘An Information Criterion’ (‘AIC’; [42]) criterion for model comparisons. The criterion comes from the likelihood theory and smaller AIC values indicate a better model. Before the DistLM analysis, we used the Draftsman plot to reduce the effect of redundant variables and examine the correlation among environmental parameters before the analyses [43]. Conductivity, the only redundant variable showing >90% correlation, was excluded from the analyses. Statistical significance (PERMANOVA, *p* < 0.05) of the DistLM routine was assessed by permutation tests where each set of samples was randomly permuted 9999 times [44]. Analyses and plots were performed using the software PRIMER v.6.1.11 [44] and RStudio v.4.3.2 [35].

To investigate pelagic food webs, we identified potential trophic relationships between plankton and small pelagic fish detected, respectively, by V9-18S data and 12S on each site in different seasons. Therefore, plankton ASVs were summed and aggregated into specific functional groups (FGs) based on their taxonomic, dimensional [45,46] and physiological similarities [3]. The use of FGs is important as it increases the representation in food web models of ecological roles played by planktonic organisms within marine ecosystems, reducing complexity and functional redundancy [19,45]. Based on this rationale, we identified in our dataset seven FGs: autotrophic protists, heterotrophic/mixotrophic protists, crustaceans, gelatinous filter feeders, jellyfish, arrow worms and terrestrial organic matter (see electronic supplementary material, tables S3 and S4).

Planktonic FGs and small pelagic fish taxa were represented in conceptual food webs considering, respectively, their relative abundance (based on reads count) and presence–absence. Putative trophic interactions among FGs and fish were obtained from the literature and GloBi [47] (see electronic supplementary material, table S5). We represented conceptual pelagic food webs using the software Gephi, v. 0.10 [26].

## 3. Results

Lagrangian particle simulations showed a higher annual average connectivity among sampling stations in the GoN. Within these latter stations, the MareChiara site was largely connected to the Sarno River mouth, while the Volturno and Sele River mouths were less connected (figure 1*b*). However, model results showed that all sites were connected to different extents, thus allowing us to intercompare biological samples and their taxonomic composition studied with eDNA metabarcoding (eDNA metaB) and interpret spatial differences in light of connectivity among sites.

We annotated 4,344 planktonic and 13 fish ASV/taxa (seven pelagic and six demersal fish ASV) using the V9-18S and 12S regions, respectively (for more details about annotation results, see electronic supplementary material, tables S3 and S6). V9-18S samples showed seasonal partitioning, with three groups of samples, the first including all winter samples and one autumn, and the second and third groups, which differed more from the first group, including the spring and summer samples, respectively (figure 1*c*).

The PERMANOVA test found significant differences in biological data among seasons (*p* < 0.05). DistLM analysis showed that environmental parameters drove biological data partitioning, with temperature, density and dissolved oxygen explaining 26.7% of the total variance (figure 1*d*). The summer group was the largest one and included two subgroups, one including samples from the inner GoN (MareChiara and Sarno stations), and one including samples from the outer GoN (Dohrn Canyon) and other gulfs’ sites. The latter observation indicates that spatial partitioning occurred, though to a lower extent than the seasonal one.

**Figure 2.**
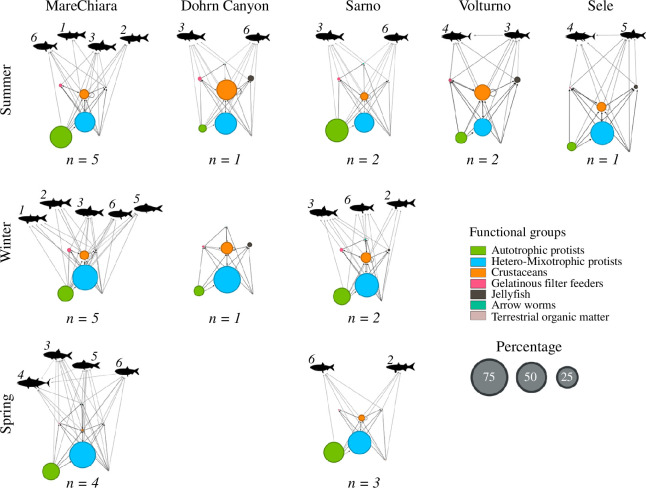
Conceptual pelagic food webs at the sampling sites in the summer, winter and spring seasons (samples collected during autumn, i.e. Nov_20 at the MareChiara station, were grouped into the winter samples). Edges represent putative trophic interactions between plankton FGs and small pelagic fish. Node size is proportional to the relative percentage of reads for V9-18S eDNA, while the information obtained from 12S (fish) was used to indicate the presence of taxa. Fish IDs: 1, *Chelon auratus*; 2, *C. labrosus*; 3, *Engraulis encrasicolus*; 4, *Euthynnus alletteratus*; 5, *Sardina pilchardus*; 6, *Sardinella aurita*. *n* is the number of samples used to calculate the percentage of total V9-18S reads.

To map compositional differences in time and space, we combined plankton FGs and small pelagic fish presence and derived conceptual pelagic food webs (figure 2). MareChiara and Sarno stations had the best spatiotemporal coverage and strong connectivity all over the year (figure 1*b*), allowing seasonal comparisons (figure 2). At both sites, heterotrophic/mixotrophic protists were predominant and more abundant in winter and spring than in summer. Autotrophic protists were higher in summer at both sites. Crustaceans, which predate the previous groups, were third in rank and found at MareChiara during summer and winter but virtually absent during spring; however, during the latter season, crustaceans were detectable at Sarno. Other FGs were weakly detected overall.

Concerning small pelagic fish communities, we also observed signals of spatiotemporal differentiation at MareChiara and Sarno. The small pelagic *S. aurita* occurred in all the seasons analysed at the MareChiara and Sarno sites, while *E. encrasicolus* was always present but not at Sarno in spring. Winter was the richest season at the MareChiara and Sarno sites, which differed for the presence of *S. pilchardus* and *C. auratus* (present only at the MareChiara site).

Dohrn Canyon samples allowed us to describe only winter and summer communities, which were markedly different from those found in the inner GoN. About plankton, autotrophic protists were scanty if compared with MareChiara and Sarno, while heterotrophic/mixotrophic protists showed similar values. Crustaceans were in higher percentage during summer and showed almost double the relative abundance of the inner GoN. The other FGs were less represented, except for jellyfish, which showed a higher relative abundance than in the inner GoN. Finally, the Dohrn Canyon and Sarno sites showed the same summer small pelagic fish community.

We also explored the spatial differences among summer communities from the GoN and the other gulfs, i.e. studied at the Volturno and Sele stations. The Volturno plankton community was similar to that present at the Dohrn Canyon but showed a higher relative number of autotrophic protists. At Sele site, heterotrophic/mixotrophic protists were the most relevant, reaching the highest relative abundance among all summer communities. Still, autotrophic protists showed a lower amount than in other coastal sites during summer. Concerning planktonic animals, Volturno was similar to the Dohrn Canyon (higher crustaceans and jellyfish than in other stations), while Sele was more similar to inner GoN stations (lower crustaceans and jellyfish). Concerning small pelagic fish, *E. alletteratus* and *E. encrasicolus* were present at both Volturno and Sele, but this taxa combination was not found in any other site during summer.

## 4. Discussion

The advance in the eDNA metaB analysis in marine ecology has profoundly increased the amount of information available on marine biodiversity. The use of eDNA presents many advantages: for instance, it is less invasive and more cost-effective than traditional surveys [48,49]. So far the eDNA technique has been mainly applied to temporal or spatial studies with only a few investigations comparing the spatiotemporal changes of marine communities at the regional scale (e.g. [50,51]).

Our study shows the importance of integrating eDNA data and connectivity analysis to understand the dynamics of coastal planktonic communities, which are carried by physical dynamics and may be moved to different sites, influencing the higher trophic levels. Our results confirmed the plankton seasonality previously described at the MareChiara station (e.g. [32,52]), but extended this observation at the whole regional scale, with the planktonic communities in the Campania coast that differed more by seasonal than by spatial dynamics. This observation is in line with a recent scientific reference about plankton we can invoke as a comparison for the study area, i.e. the observation that the genetic fingerprint of populations of the diatom species *Pseudo-nitzschia multistriata* collected in the different Campania gulfs was spatially very similar, but far different over time [53].

Environmental factors determined these seasonal differences. Summer and winter communities detected with V9-18S eDNA metaB were mainly influenced by temperature and density, respectively, which remark the water column stratification cycles affecting nutrient availability in the photic zone influencing the metabolism, growth, reproduction, development, distribution and food availability of marine organisms [54,55]. The higher presence of phototrophs in summer and of heterotrophic/mixotrophic protists in winter we observed in the GoN matched that from a longer metabarcoding time series from the MareChiara site [32]. Spring communities are also influenced by dissolved oxygen (DO), which is regulated by both biotic (e.g. production and consumption), and abiotic factors (e.g. pH, temperature, salinity and hydrodynamic processes) [56,57]. DO concentrations can lead to changes in planktonic community structure and trophic transfer [58,59], with crustacean zooplankton peaking in hypoxic waters where they seek refuge from predation by fish, which instead exhibit lower abundance at lower oxygen levels [60,61].

**Figure 3.**
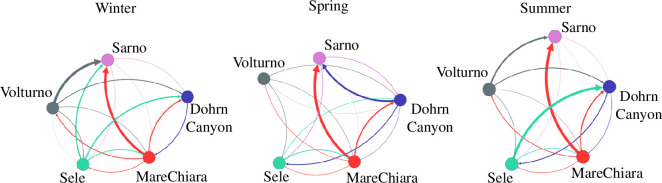
Connectivity network. Network edges represent connections between sites. Edges directed from release to the arrival site represent the particle release and are colour-coded by the release site. Edge width is proportional to the sum of the values of particle migration rate, grouped by the sampling site’s area of interest.

Most of the spatial homogeneity that we detected could be the result of high connectivity at the regional scale (figure 3). For instance, the strong connection between the Volturno and GoN areas matches previous modelling studies’ results [62,63].

However, spatial differentiation can also emerge from the multiple stimuli that local environmental conditions may exert on the pelagic communities, which are highly dynamic entities. The tangled action of species immigration and local selection can affect spatial differentiation among communities and their food webs’ functioning during the same season, like summer.

As a general pattern, phototroph-driven food webs and higher fish diversity occurred at eutrophic stations closer to the coast undergoing stronger inputs from land runoff and showing higher retention times, like in the inner GoN (MareChiara and Sarno), suggesting a more direct flux of matter from primary producers to higher consumers. Conversely, food webs at offshore stations (Dohrn Canyon), or those more exposed to the action of open sea currents (Volturno and Sele), showed a lower dependence on strictly phototrophic plankton, higher prevalence of heterotrophic/mixotrophic protists adapted to oligotrophic conditions, and less diverse small pelagic fish communities, suggesting a more dissipative microbial loop-based food web with longer trophic chains [64]. Oligotrophic sites also included more jellyfish, which compete with planktivorous fish [65], probably owing to upwelling transporting specimens from deeper waters [66].

Overall, we observed that slightly different plankton assemblages co-occurred with the different small pelagic fishes (figure 2) showing mainly a planktivorous diet composed of copepods, and to a lesser extent other crustaceans, molluscs, pelagic tunicates and other fishes [67,68]. The assembly of small pelagic fishes can be influenced by several interplaying drivers, such as food quality [13,14], biological (e.g. spawning timing) and environmental (e.g. salinity tolerance) factors [15,69].

Concerning biological factors, small pelagic fishes show different spawning times, which may have affected the occurrence of fish DNA in our study. Sardine (*S. pilchardus*, #5 in figure 2) spawns during autumn–winter in a mixed water column with salinities around 37–38 psu [68,69], which corresponds with the environmental conditions that we retrieved. Other species have their optimum reproduction and spawning during spring and summer when the water column is stratified and warmer, as in the case of skipjack tuna (*E. alletteratus*; #4) (23–27.5°C) and anchovy (*E. encrasicolus*; #3) (13–25.5°C) [70], which can spawn in a wide range of salinity (36.7–37.9 and 29.1–38.2 psu, respectively) [70,71]. Sardinella (*S. aurita*; #10) also spawns at temperatures above 23°C, from July to October in agreement with its tropical origin [69].

Regarding environmental factors, salinity tolerance can also drive fish assemblages. For instance, we found *C. auratus* (#1) only at MareChiara station, and *C. labrosus* (#2) also at the River Sarno mouth. This is in agreement with observations of *C. auratus* having mainly a pelagic behaviour, while *C. labrosus* is euryhaline and frequently found in estuaries [72]. The genus *Chelon* includes generalist planktivorous–detritivorous fish [73,74] often found around sea bream and sea bass farms [72,75] and polluted environments [76].

However, we must consider that the small pelagic fish species found in our survey may not reflect the entire diversity present in the study area. Indeed, fishes are characterized by a scattered spatial distribution [70]; water sampling can fail to catch all the taxa retrieved from traditional approaches, such as visual census and fishing nets [49,50]; eDNA shows operational limits like primer specificity [50], and it cannot provide information on the true abundances of organisms [47,48].

## 5. Conclusion

The application of eDNA metabarcoding analysis in marine ecology has expanded our understanding of the spatiotemporal dynamics of marine biodiversity at the regional scale. By applying an integrative approach, this study highlighted the importance of combining eDNA data with connectivity analyses to reveal the complex dynamics of coastal planktonic communities, which are influenced by physical processes and can spread between different sites affecting, in this way, the higher trophic levels, like fishes. In this respect, our study highlights the need to further investigate the intricate interactions that regulate coastal marine communities and their underlying ecological processes, by intensifying the fish eDNA sampling effort at the spatial level and integrating these observations with traditional approaches like fishery surveys, which could provide more precise information on the distribution of fish in the water column. Such knowledge is critical for the effective conservation and management of marine ecosystems in the face of environmental change.

## Data Availability

The datasets supporting this article have been uploaded as part of the electronic supplementary material [77]. Raw V9-18S and 12S metabarcoding data are available in the Sequence Read Archive (SRA) under BioProject PRJNA1023387.

## Dedication

The authors dedicate this work to the memory of Prof. Ramon Margalef on the 20th anniversary of his passing and because of his permanent and continued inspiration towards ecological community studies at the intersection of biological and physical disciplines.
